# Role of FOXM1 and AURKB in regulating keratinocyte function in psoriasis

**DOI:** 10.1515/med-2024-1049

**Published:** 2024-09-30

**Authors:** Zhaofeng Zhao, Jie Cheng, Qiang Hou, Jian Zhu, Tu Chen, Sheng Lu, Guiju Wu, Hongli Lv, Xiujuan Wu

**Affiliations:** Central Laboratory, Shanghai Xuhui Central Hospital, Zhongshan-Xuhui Hospital, Fudan University, Shanghai, 200031, P.R. China; Department of Urology, Shanghai Xuhui Central Hospital, Zhongshan-Xuhui Hospital, Fudan University, Shanghai, 200031, P.R. China; Department of Dermatology, Xuhui District Dahua Hospital, Shanghai, 200237, P.R. China; Department of Dermatology, Shanghai Xuhui Central Hospital, Zhongshan-Xuhui Hospital, Fudan University, Shanghai, 200031, P.R. China; Department of Dermatology, Changqiao Street Community Health Service Center, Shanghai, 200231, P.R. China; Department of Dermatology, Jia Ding Central Hospital, No. 01, Dingcheng Road, Jiading District, Shanghai, 201899, P.R. China; Department of Dermatology, Shanghai Xuhui Central Hospital, Zhongshan-Xuhui Hospital, Fudan University, No. 366, Longchuan North Road, Xuhui District, Shanghai, 200031, P.R. China

**Keywords:** FOXM1, AURKB, psoriasis, HaCaT cells, proliferation, cycle

## Abstract

**Objective:**

This study investigated the effect of forkhead box M1 (FOXM1) and Aurora kinase B (AURKB) on the epidermal function of keratinocytes.

**Methods:**

Bioinformatics analysis was used to analyze the co-expression network of FOXM1 and its correlation with AURKB. The expression of FOXM1 and AURKB in tissues and cells was detected by immunofluorescence and real-time quantitative polymerase chain reaction, respectively. HaCaT cells were transfected with si-FOXM1 to knock down FOXM1. Cell proliferation was detected by cell counting kit-8 assay. Cell migration was detected by scratch assay. Cell invasion was detected by the Transwell invasion assay. Cell apoptosis and cell cycle were detected by flow cytometry.

**Results:**

FOXM1 and AURKB were positively correlated and highly expressed in psoriatic lesions. After transfection of si-FOXM1, the expression levels of FOXM1 and AURKB genes significantly decreased. The proliferation of HaCaT cells decreased, the apoptosis rate increased significantly, and the proportion of cells in the G1 phase increased significantly, while the proportion of cells in the S phase decreased significantly. The scratch closure of HaCaT cells was reduced, and the number of cell invasions decreased significantly.

**Conclusion:**

FOXM1 and AURKB may affect the progression of psoriasis by regulating the proliferation, cell cycle, migration, and invasion of keratinocytes.

## 1 Introduction

Psoriasis is a chronic inflammatory skin condition characterized by the uncontrolled proliferation of keratinocytes and impaired differentiation, leading to the formation of thick, scaly plaques on the skin [1,2]. Keratinocytes play a crucial role in maintaining the skin barrier function and homeostasis. Under normal conditions, they differentiate from basal cells in the epidermis, progressing through multiple layers and stages of differentiation to eventually form the stratum corneum. In psoriasis, there is impaired differentiation of keratinocytes, where keratinocytes lose their ability to differentiate in a controlled manner [3]. Moreover, dysregulated differentiation leads to the accumulation of hyperproliferative keratinocytes, epidermal hyperplasia, and the formation of psoriatic plaques. Additionally, the altered differentiation process contributes to the inflammatory response in the skin, further exacerbating the disease [3]. In psoriatic lesions, inflammatory signals trigger an abnormal immune response within the skin and result in the increased production of cytokines. These cytokines may lead to excessive proliferation of keratinocytes and disrupt the normal process of differentiation, which in turn induces the production of a large amount of pro-inflammatory cytokines, amplifying the inflammatory response and leading to the development of psoriasis [4,5]. There are various cytokines in the tissues of psoriasis patients, including IL-23, tumor necrosis factor-alpha (TNF-α), interferon-γ, IL-1α, IL-1β, IL-6, IL-8, VEGF, and TGF-α1 [4]. IL-6, together with TGF-β produced by keratinocytes, induces naïve CD4+ T cells to differentiate into Th17 cells [5,6]. Additionally, IL-23 stimulates the activation of Th17 cells, leading to the secretion of IL-17A and IL-22 [7]. Despite the advancement of targeted therapies, psoriasis remains a treatable but incurable disease. In particular, the inhibition of IL-23 and IL-17 has shown significant clinical efficacy in targeted therapies. Although numerous targeted therapeutic options have been identified in studies, further research is required to investigate the regulation of the forkhead box M1 (FOXM1) pathway and its targeting mechanisms in psoriasis.

The FOXM1 is a transcription factor within the Forkhead box protein superfamily, and it serves as a pivotal regulator of cell proliferation [8]. FOXM1 modulates the production of IL-7, IL-6, IL-23, and TGF-β [9,10]. Knockdown of FOXM1 suppresses the production of IL-6, IL-23, and TGF-β induced by TNF-α in HaCaT keratinocytes, which may help prevent the activation of Th17 cells [7]. Moreover, FOXM1 impacts various biological processes, such as cell cycle, cell proliferation, renewal, pluripotency, differentiation, DNA damage repair, tissue homeostasis, migration, angiogenesis, and survival [11,12,13,14]. It can directly interact with Skp2, Cks1, cyclin A, and cyclin B, which are essential for mediating the cell cycle [12,13]. FOXM1 is implicated in all hallmarks of human cancer, and its overexpression is correlated with the invasiveness and low survival rates of specific solid tumors [15]. Multiple studies [16,17] have demonstrated that FOXM1 manifests its pro-carcinogenic activity through diverse downstream targets. FOXM1 is highly expressed in squamous epithelial carcinoma, promoting the activation of the MYC gene and loss of the p53 gene, resulting in the abnormal proliferation of tumor epithelial cells [18]. In melanoma, inhibitors that effectively target the high expression of intracellular FOXM1 have been shown to weaken the proliferative ability of melanoma cells [19]. Therefore, the identification of FOXM1 targets and their specific roles in various human cancers holds the utmost importance in cancer management.

FOXM1 activates the transcription and expression of various targets, including aurora kinase A and aurora kinase B (AURKB), directly and indirectly, and interacts with various signaling pathways such as the FOXM1/AKT-positive feedback loop, Wnt/β-catenin, TGF-β/SMADs, and STAT3, participating in cellular physiological and pathological processes [8,16,20,21]. FOXM1 has also been found to influence skin diseases and regulate the proliferation and apoptosis of keratinocytes [7]. However, the exact mechanism of action remains unclear. FOXM1 is highly expressed in psoriatic lesional skin tissues. Its knockdown inhibits cell proliferation and promotes apoptosis, while its overexpression promotes cell proliferation and suppresses apoptosis of TNF-α stimulated HaCaT keratinocytes [7]. Additionally, FOXM1 mediates the regulation of TNF-α-induced cell proliferation, apoptosis, and inflammation in HaCaT cells via the NF-κB pathway [7]. Through the regulation of TNF-α-induced proliferation, apoptosis, and inflammation in keratinocytes, FOXM1 promotes psoriasis [11]. Targeting FOXM1, its downstream targets, and the associated signaling pathways presents a novel approach for the development of efficacious anti-cancer drugs.

AURKB belongs to the subfamily of aurora kinases, which encode serine/threonine kinases. These kinases regulate the alignment and segregation of chromosomes during cellular mitosis and meiosis by binding to microtubules [22]. It has been reported that inhibiting AURKB can reduce cell viability and induce apoptosis in melanoma cells [23]. AURKB can enhance the proliferation of gastric cancer cells both *in vivo* and *in vitro* [24]. Silencing AURKB expression can inhibit gastric cell proliferation and prevent cell cycle progression into the G2/M phase [24]. Additionally, AURKB also plays a crucial role in gastric cancer cell proliferation by activating the expression of cyclin D1 through phosphorylation of H3S10 in the cyclin D1 promoter [24]. Currently, there are no published research findings available regarding the mechanism of action associated with AURKB. However, it has been observed that AURKB triggers the activation of the AKT/mTOR pathway. Notably, the inflammatory vesicle activation induced by overexpressed AURKB was found to be attenuated by an Akt inhibitor (PI-103). According to recent research, AURKB has been shown to promote inflammation associated with psoriasis by obstructing the autophagy-mediated inhibition of AIM2 inflammatory vesicles [25].

Herein, we investigated the role and mechanism of FOXM1 and AURKB in keratinocytes. The expression levels of FOXM1 and AURKB were detected. The FOXM1 gene was knocked down in keratinocytes. The cellular functions such as cell proliferation, cell cycle, and colony formation in keratinocytes were then assessed. Our findings may provide a new perspective on the pathogenesis of psoriasis, associated pathways, and potential therapeutic approaches.

## 2 Methods

### 2.1 Skin sample collection

The psoriatic lesional skin tissues were collected from five patients with plaque psoriasis in the Department of Dermatology, Xuhui Central Hospital, Shanghai, from January 2022 to June 2022. Normal skin tissues from the surrounding area were included as controls. All enrolled patients were diagnosed with psoriasis vulgaris by a dermatologist. They had not received treatment with antibiotics, probiotics, glucocorticoids, or immunosuppressants in the past 6 months. Additionally, they did not have gastrointestinal or immune disorders or any other serious illnesses.

### 2.2 Bioinformatics analysis

Bioinformatics analysis was conducted using the RNA sequencing data of psoriatic lesional skin tissue and normal skin tissue from our previous study [26]. The correlation between FOXM1 and the differentially expressed genes of disease versus normal was analyzed using Pearson correlation analysis. Significant correlation was defined using a significance threshold (*P* < 0.05) and correlation coefficient threshold (|*r*| > 0.5). A co-expression network was constructed using Cytoscape (https://cytoscape.org/). Metascape (v3.5.20230501) was used for GO and KEGG functional enrichment analysis of the mRNAs in the co-expression network. The enriched terms at the levels of biological processes, molecular functions, and cellular components, as well as the functional pathways of these genes, were analyzed.

### 2.3 Immunofluorescence staining

The skin tissues were fixed in 10% formalin, embedded in paraffin, and prepared into 5 μm sections. The sections were then incubated with primary antibodies of anti-FOXM1 (Bioss Biotech, Beijing, China) and anti-AURKB (Bioss Biotech) at 4°C overnight. After washing with phosphate-buffered saline, incubation with Goat Anti-Rabbit fluorescent secondary antibodies (Bioss Biotech) was conducted at room temperature for 1 h. Finally, the images were observed under an Olympus CKX53 fluorescence microscope (Olympus, Tokyo, Japan).

### 2.4 Cell culture and transfection

The human epidermal keratinocyte (HaCaT) cells were obtained from the Cell Bank of the Chinese Academy of Sciences. They were cultured with DMEM containing 10% fetal bovine serum at 37°C in a 5% CO_2_ cell incubator. When the cell confluency reached approximately 70%, si-NC (negative control) and si-FOXM1 (GenePharma) were separately transfected into HaCaT cells using Hilymax (BD, San Jose, CA, USA). The sequence for si-FOXM1 was sense: 5′-GGUGACUUCAAGAUCAAAUTT-3′ and antisense: 5′-AUUUGAUCUUGAAGUCACCTT-3′. At 48 h after transfection, the cells were collected for subsequent experiments.

### 2.5 Real-time quantitative polymerase chain reaction (PCR)

After 48 h of cell transfection, the cells from each group were collected. Total RNA from each group of cells was extracted using TRIzol reagent (Vazyme Biotech Co., Ltd, Nanjing, China). Following the instructions of the reverse transcription kit (Vazyme Biotech) and the fluorescence real-time quantitative PCR kit (Vazyme Biotech), RNA was reverse transcribed into cDNA and subjected to PCR, with GAPDH as the internal reference. The primer sequences are presented in supplementary Table A1. The PCR procedures were 95°C for 3 min and 40 cycles of 95°C for 5 s and 60°C for 30 s. The relative gene expression was calculated with the 2^−ΔΔCt^ method.

### 2.6 Cell counting kit-8 (CCK-8) assay

Cell proliferation was measured using the CCK-8 cell proliferation assay kit (Dojindo, Tokyo, Japan). HaCaT cells in the logarithmic growth phase were seeded at a density of 4,000 cells per well in a 96-well plate. After transfection with si-NC or si-FOXM1, cells were incubated for 0, 24, 48, and 72 h. Then, 10 μL of CCK-8 reagent was added to each well and incubated at 37°C for 2 h. The OD values of each group were measured at a wavelength of 450 nm using a microplate reader.

### 2.7 Flow cytometry analysis

For detecting cell apoptosis, after transfection for 48 h, cells were collected and re-suspended to a cell density of 1 × 10^6^ cells/mL. Then, 100 μL of cell suspension was stained with Annexin V-FITC/PI following the instructions of the apoptosis detection kit (BD). Cell apoptosis rate in each group was detected on a flow cytometer.

For detecting the cell cycle, cells were collected after 48 h of transfection and fixed in pre-cooled 70% ethanol overnight. Based on the instructions of the cell cycle detection kit (BD), fixed cells in each group were stained with PI/Rnase A. Subsequently, the flow cytometer was used to analyze the cell cycle distribution in each group.

### 2.8 Scratch assay

HaCaT cells in the logarithmic growth phase were seeded in a 24-well plate and transfected accordingly. When the cell confluence reached over 90%, a scratch was made on the surface of the seeded cells using a 10 μL pipette tip, and the scratch was properly marked. Photographs were taken at 0 and 24 h after the scratch to observe the healing of the scratch. ImageJ software was utilized to calculate the scratch closure rate.

### 2.9 Transwell invasion assay

After transfection, cells from each group were collected and resuspended with a serum-free medium. The cell concentration was then adjusted to 2.5 × 10^5^ cells/mL. 200 μL of cell suspension was added to the upper chamber of the Transwell (Millipore), which was pre-coated with a basement membrane gel. Then, 500 μL of complete culture medium was added to the lower chamber. The Transwell was incubated at 37°C and 5% CO_2_ for 24 h. After removing the cells in the upper chamber with a cotton swab, they were fixed with paraformaldehyde and stained with crystal violet. The invaded cells were then counted under a microscope.

### 2.10 Statistical analysis

Statistical analysis was performed using SPSS 20.0 software. All data are expressed as mean ± standard deviation. The *t*-test was used to compare the differences between the two groups, with *P* < 0.05 indicating statistical significance.


**Ethics approval:** This study protocol was reviewed and approved by the Institutional Ethics Committee of Shanghai Xuhui Central Hospital.
**Informed consent:** All patients were informed about the study and gave their consent.

## 3 Results

### 3.1 Bioinformatics analysis results

**Figure 1:**
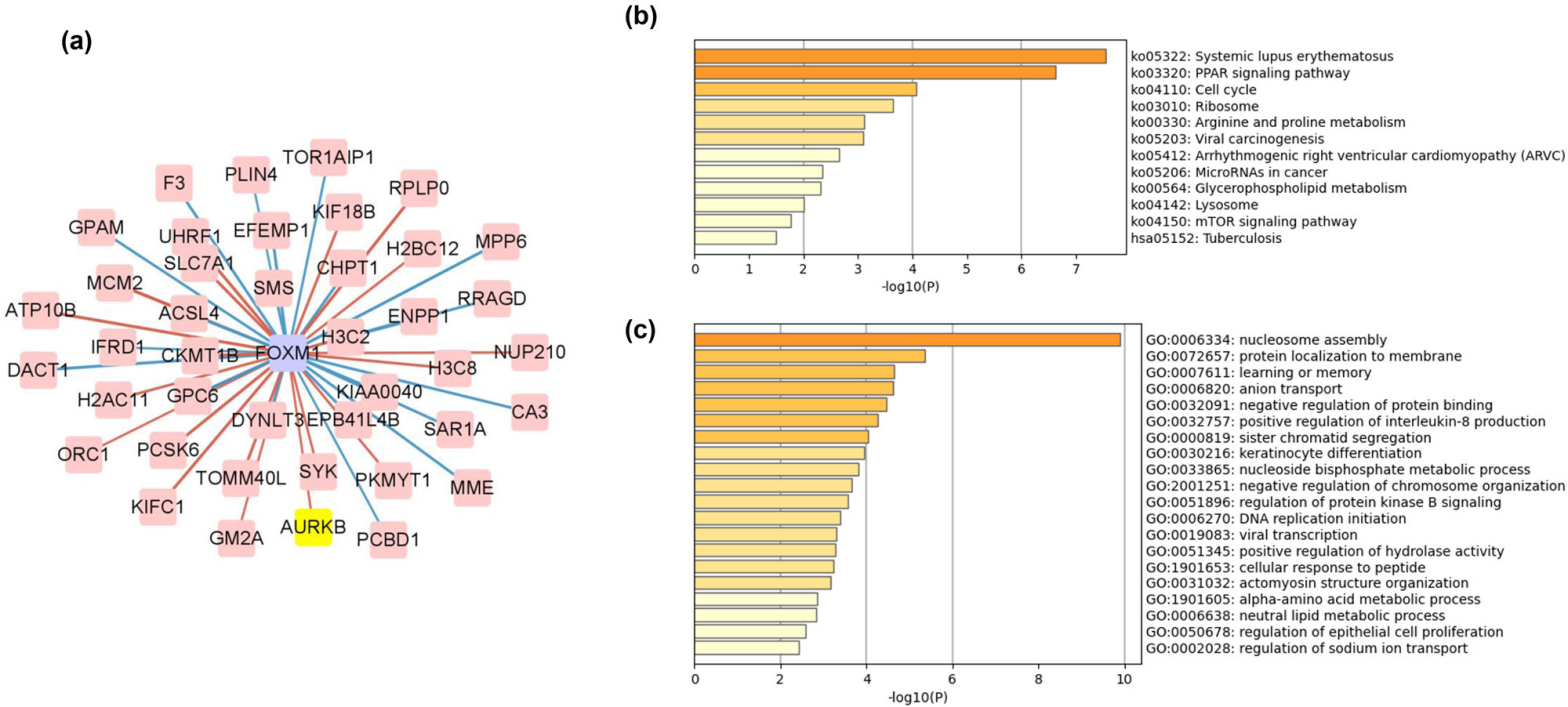
Bioinformatics analysis of FOXM1 and differential genes related to psoriasis. (a) Co-expression network of FOXM1 and differential genes of psoriasis. Pink lines represent a positive correlation, while blue lines represent a negative correlation. (b) KEGG pathway analysis of the differential genes of psoriasis. (c) GO functional clustering of the differential genes of psoriasis.

To determine the regulatory role of FOXM1 in psoriasis, we conducted a correlation analysis between FOXM1 and differentially expressed genes in psoriasis. The results showed that in the psoriatic tissues, FOXM1 was positively correlated with genes such as AURKB, KIFC1, and UHRF1 and negatively correlated with genes such as PLIN4, RRAGD, and MME (Figure 1a). These differentially expressed genes were mainly enriched in functional pathways such as the cell cycle, mTOR signaling pathway, and regulation of epithelial cell proliferation, as shown in Figure 1b and c. Based on this, it can be inferred that FOXM1 may regulate the progression of psoriasis by modulating these differentially expressed genes, thereby regulating the cell cycle and psoriasis progression.

### 3.2 FOXM1 and AURKB expression levels are up-regulated in psoriatic tissue

**Figure 2:**
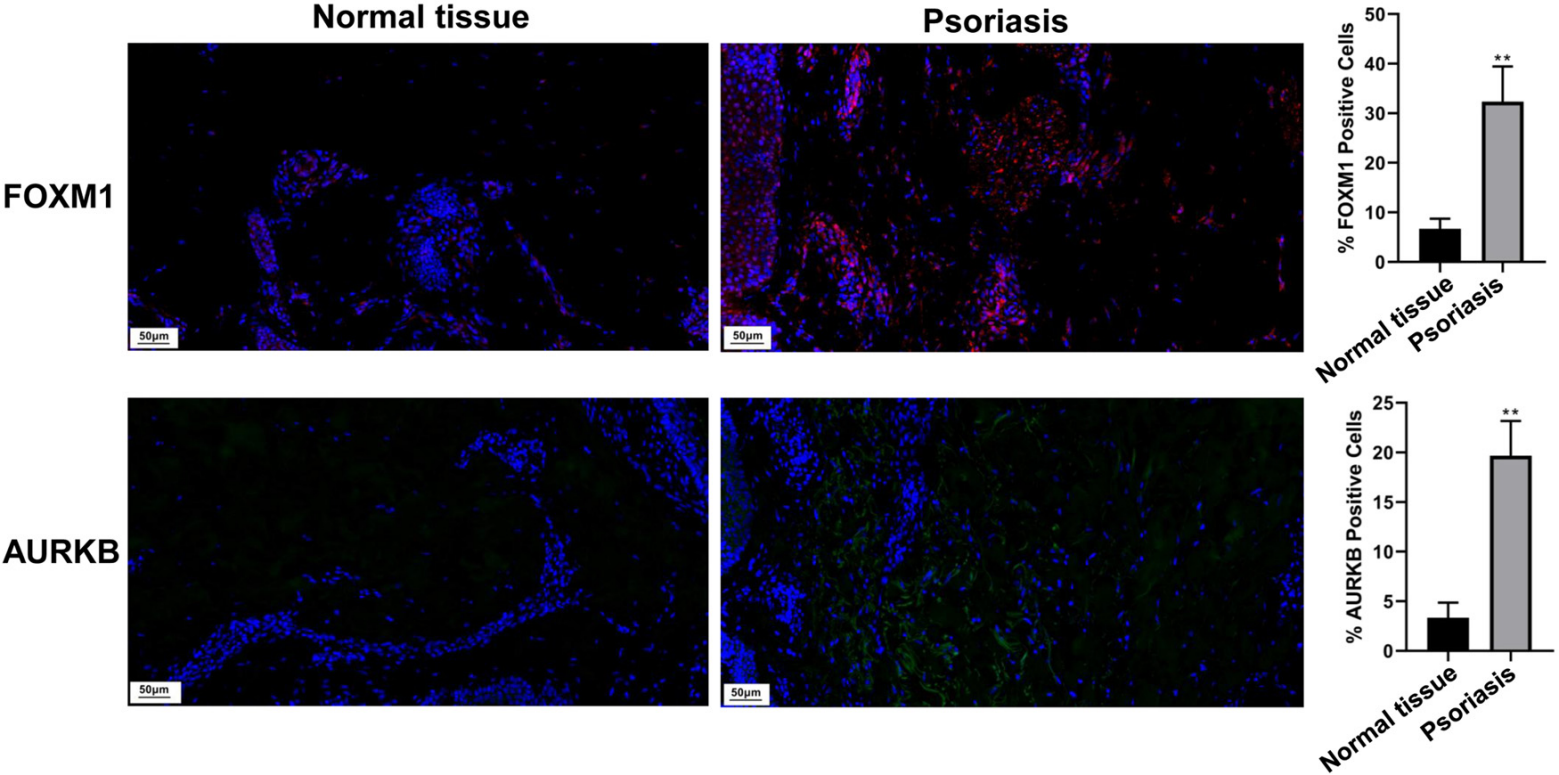
Analysis of FOXM1 and AURKB expression. Immunofluorescence staining was used to detect FOXM1 and AURKB expression in psoriatic lesional skin tissues (*n* = 5) and normal skin tissues (*n* = 5) (scale bar = 50 μm). The percentage of FOXM1/AURKB-positive cells was compared. Compared with NC, **P < 0.01.

To determine the expression of FOXM1 and AURKB in the psoriatic lesional skin tissues and normal skin tissues, we performed immunofluorescence staining. The results revealed an increase in the expression of FOXM1 and AURKB in the lesional skin of psoriasis patients compared to the normal skin tissues (Figure 2). These findings suggest that alterations in the expression of FOXM1 and AURKB may play a crucial role in the development and progression of psoriasis.

### 3.3 The expression of *FOXM1* and *AURKB* in HaCaT cells after FOXM1 knockdown

Real-time quantitative PCR revealed that after transfection of HaCaT cells with si-FOXM1 for 48 h, the expression of *FOXM1* and *AURKB* genes was significantly inhibited, compared to the si-NC group (*P* < 0.05) (Table 1). This indicates that si-FOXM1 not only significantly inhibits the expression of the *FOXM1* gene in HaCaT cells but also indirectly inhibits the expression of the *AURKB* gene.

**Table 1:** The expression of *FOXM1* and *AURKB* in HaCaT cells after FOXM1 knockdown

Group	FOXM1	AURKB
si-NC	1.02 ± 0.27	1.01 ± 0.13
si-FOXM1	0.24 ± 0.09	0.56 ± 0.12
*t*	4.851	4.382
*P*	0.008	0.012

### 3.4 The proliferation of HaCaT cells decreases after FOXM1 knockdown

Following si-FOXM1 transfection in HaCaT cells, the proliferation visibly declined over time, as detected by CCK-8 assay. Compared to the si-NC control group, the OD450 value of the si-FOXM1 group was significantly lower at 24, 48, and 72 h, indicating significantly slower cell proliferation rates after FOXM1 knockdown (*P* < 0.05) (Table 2). This suggests that inhibiting the expression of FOXM1 can suppress the proliferation of HaCaT cells.

**Table 2:** Effect of FOXM1 knockdown on proliferation of HaCaT cells

Group	OD (450 nm)			
	0 h	24 h	48 h	72 h
si-NC	0.52 ± 0.03	0.75 ± 0.03	1.30 ± 0.08	1.88 ± 0.09
si-FOXM1	0.53 ± 0.02	0.66 ± 0.04	1.03 ± 0.10	1.36 ± 0.11
*t*	0.339	3.046	3.867	6.470
*P*	0.752	0.038	0.018	0.003

### 3.5 FOXM1 knockdown increases cell apoptosis and inhibits cell cycle progression

**Figure 3:**
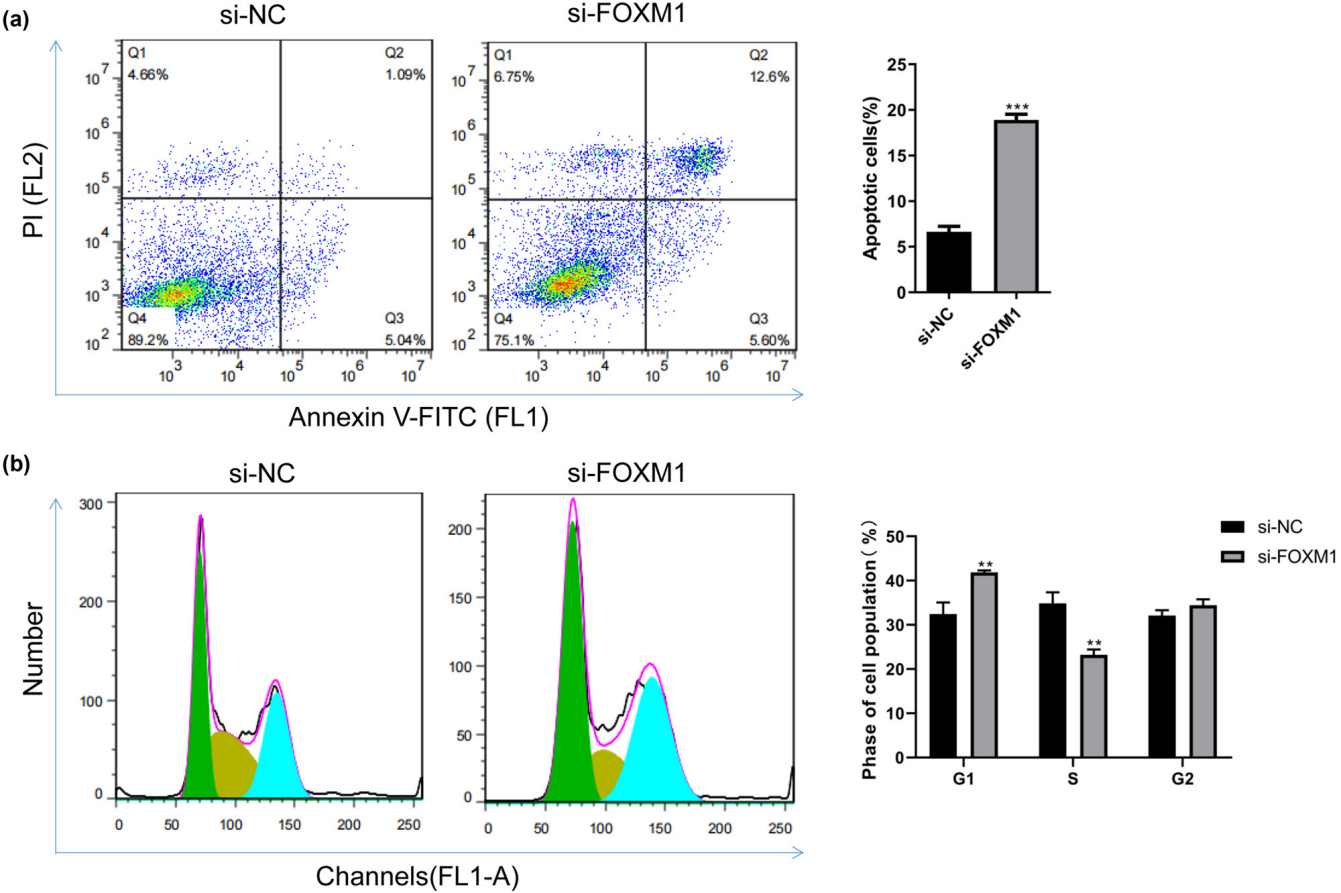
The effect of inhibiting FOXM1 on apoptosis and cell cycle of HaCaT cells. (a) Flow cytometry analysis of apoptosis in HaCaT cells after transfection with si-FOXM1. Cell apoptosis rate was compared. (b) Flow cytometry analysis of cell cycle in HaCaT cells after transfection with si-FOXM1. The percentage of cells in each cell cycle phase was compared. Compared with si-NC, **P < 0.01, ***P < 0.001.

To investigate the effects of FOXM1 on apoptosis and the cell cycle of keratinocytes, we transfected si-FOXM1 into HaCaT cells for 48 h and performed flow cytometry analysis. The results showed that compared to the si-NC group, the si-FOXM1 group exhibited a significant increase in the level of cell apoptosis in HaCaT cells (Figure 3a). Additionally, the si-FOXM1 group showed a significantly higher proportion of cells in the G1 phase and a significantly lower proportion of cells in the S phase (*P* < 0.05) (Figure 3b). These findings demonstrate that FOXM1 can modulate the apoptosis and cell cycle of keratinocytes.

### 3.6 FOXM1 knockdown inhibits the migration and invasion of HaCaT cells

**Figure 4:**
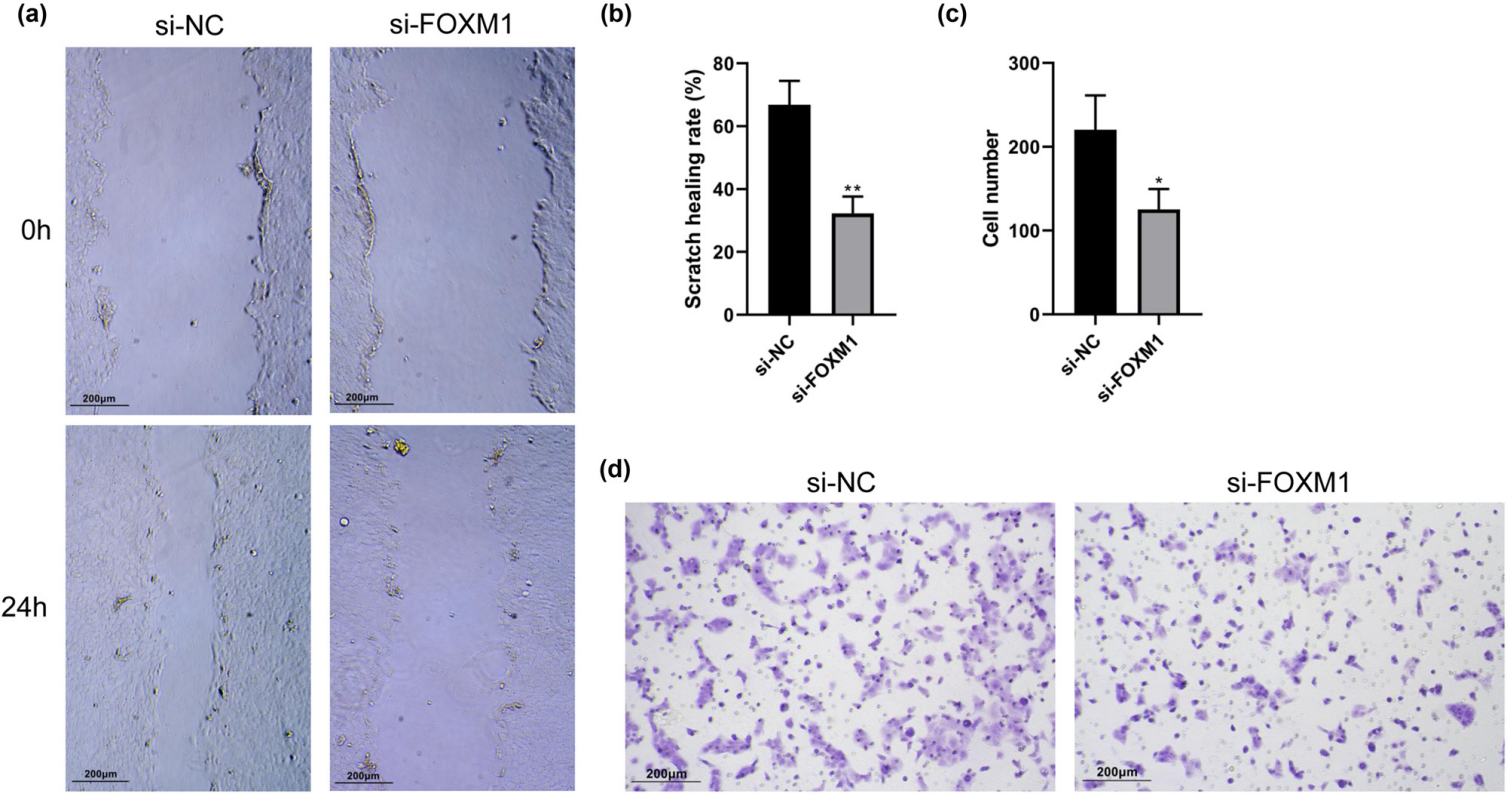
The effect of inhibiting FOXM1 on migration and invasion of HaCaT cells. (a) Scratch assay to detect the migration of HaCaT cells after transfection with si-FOXM1. (b) Scratch healing rate. (c) Number of invaded cells. (d) Transwell invasion assay to detect the invasion of HaCaT cells after transfection with si-FOXM1. Compared with si-NC, *P < 0.05, **P < 0.01.

After si-FOXM1 transfection, the migration and invasion abilities of HaCaT cells were assessed by Scratch assay and Transwell invasion assay, respectively. As shown in Figure 4a and b, the scratch closure rate after scratching was significantly suppressed in the si-FOXM1 group compared to the si-NC group (*P* < 0.05). Transwell invasion assay results showed a significant decrease in the number of cells that passed through the Transwell chamber membrane in the si-FOXM1 group compared to the si-NC group (*P* < 0.05) (Figure 4c and d). This suggests that the migration and invasion functions of HaCaT cells are significantly suppressed after inhibiting FOXM1 expression.

## 4 Discussion

Psoriasis can be triggered by various factors such as infection, trauma, and medication, with the main pathological changes being abnormal proliferation of epidermal keratinocytes and incomplete keratinization [27]. The specific causes of this disease remain unclear and controversial, but benign overgrowth of keratinocytes is one of its main features, along with immune system abnormalities [28]. Since the excessive proliferation of keratinocytes is one of the main characteristics of psoriasis, it is crucial to study the changes in cellular functions of keratinocytes during the progression of psoriasis and identify the factors causing these changes. The effect of FOXM1 and AURKB on the epidermal function of keratinocytes was the focus of this study. Utilizing bioinformatics analysis, we examined the co-expression network of FOXM1 and its relationship with AURKB. Immunofluorescence and real-time quantitative PCR analyses were employed to assess the expression levels of FOXM1 and AURKB in tissues and cells. Knockdown of FOXM1 was achieved by transfecting HaCaT cells with si-FOXM1, leading to a significant decrease in the expression levels of both FOXM1 and AURKB genes. The findings revealed a positive correlation between FOXM1 and AURKB, with high expression levels observed in psoriatic lesions. Following knockdown of FOXM1, a notable decrease in cell proliferation, increased apoptosis, alterations in cell cycle distribution, reduced cell migration, and decreased cell invasion were observed. These results suggest that FOXM1 and AURKB may play crucial roles in regulating the progression of psoriasis by affecting the proliferation, cell cycle dynamics, migration, and invasion of keratinocytes.

A study [29] found that FOXM1 and its target genes PLK1 and AURKB were often overexpressed in esophageal cancer and exhibit cross-regulation and interaction, collectively affecting the cell cycle, proliferation, and migration of esophageal cancer cells. Therefore, we speculate that the changes in the proliferation and cell cycle of keratinocytes may be related to the FOXM1–AURKB signaling pathway. In this study, through bioinformatics analysis, we found that compared to normal skin tissues, the expression levels of FOXM1 and AURKB were significantly increased in psoriasis lesions. Moreover, FOXM1 and AURKB were positively correlated. Additionally, we performed immunofluorescence on psoriasis lesions and found elevated expression levels of FOXM1 and AURKB in the lesions. These results suggest that FOXM1 and AURKB may be involved in the progression of psoriasis.

Previous studies [30,31] have demonstrated that FOXM1 plays a pivotal role as a key regulatory gene in the pathogenesis of multiple diseases by regulating genes related to proliferation, the cell cycle, migration, and apoptosis, as well as genes associated with diagnosis, treatment, and tissue repair [11]. FOXM1 has been identified as a transcription factor activated in psoriasis among the differentially expressed genes related to psoriasis and interacts with psoriasis-responsive elements to regulate cell proliferation and promote cell cycle progression during the G1/S and G2/M transitions [32,33]. In our study, we knocked down FXOM1 in HaCaT cells by using siFOXM1. The effect of FXOM1 on the expression of AURKB and the cellular functions, including proliferation, apoptosis, cell cycle, migration, and invasion of keratinocytes were evaluated. Our findings revealed that siFOXM1 downregulated the expression of FOXM1 and AURKB genes in HaCaT cells. Moreover, cell proliferation significantly decreased, apoptosis rate significantly increased, and the proportion of cells in the G1 phase exhibited an increase, while the proportion of cells in the S phase decreased concurrently. Additionally, the migratory and invasive capacities of the cells markedly declined. These results are consistent with previous research [34], suggesting the role of FOXM1 in cell cycle regulation and cell proliferation. These findings indicate a reciprocal regulatory interaction between FOXM1 and AURKB, and suppression of FOXM1 expression can inhibit the proliferation, migration, and invasion abilities of HaCaT cells while promoting apoptosis.

In conclusion, through bioinformatics analysis and experimental validation, we identified a positive correlation between FOXM1 and AURKB expression, with both genes elevated in psoriatic lesions, implying their potential involvement in psoriasis pathogenesis. Moreover, the knockdown of FOXM1 resulted in decreased cell proliferation, increased apoptosis, and altered cell cycle distribution, suggesting a key role for FOXM1 in regulating keratinocyte growth and survival. Additionally, reduced cell migration and invasion capabilities following FOXM1 knockdown indicate the importance of FOXM1 in facilitating keratinocyte mobility and tissue infiltration. Our findings suggest the potential of FOXM1 and AURKB as therapeutic targets in psoriasis by modulating keratinocyte proliferation, cell cycle progression, migration, and invasion, thus offering new avenues for future research and treatment strategies in psoriasis management. Moving forward, our future research will investigate the downstream signaling pathways influenced by FOXM1 and AURKB in keratinocytes to gain insights into the molecular mechanisms underlying their effects on cell functions. Moreover, we will conduct *in vivo* studies using animal models to validate the findings observed in cell-based experiments and evaluate the effects of FOXM1/AURKB modulation on psoriasis development and progression.

## Appendix

**Table A1:** The primer sequences for real-time quantitative PCR

Gene	Upstream	Downstream
*FOXM1*	5′-GCAGCGACAGGTTAAGGTTGAG-3′	5′-GGAAAGGTTGTGGCGGATG-3′
*AURKB*	5′-AGCGAACAGCCACGATCAT-3′	5′-ACCAGCCGAAGTCAGCAAT-3′
*GPADH*	5′-TCAAGGCTGAGAACGGGAAG-3′	5′-CGCCCCACTTGATTTTGGAG-3′
